# Daily supplementation of lesser mealworm protein for 11-weeks increases skeletal muscle mass in physically active older adults

**DOI:** 10.1016/j.jnha.2024.100364

**Published:** 2024-09-21

**Authors:** Lotte Koopmans, Marcia Spoelder, Coen C.W.G. Bongers, Thijs M.H. Eijsvogels, Maria T.E. Hopman

**Affiliations:** a Department of Medical BioSciences, Integrative and Exercise Physiology Research Groups, Radboud University Medical Center, Nijmegen, the Netherlands; b Department of Primary and Community Care, Radboud University Medical Center, Nijmegen, the Netherlands; c School of Sport and Exercise, HAN University of Applied Sciences, Nijmegen, the Netherlands

**Keywords:** Elderly, *Alphitobius diaperinus*, Body composition, Sarcopenia, Insect

## Abstract

**Background:**

Adequate protein intake is important to maintain skeletal muscle mass in older adults and to prevent sarcopenia. Insect-based supplements were recently introduced to the market as an environmentally friendly protein alternative. We examined the effect of daily supplementation of lesser mealworm (*Alphitobius diaperinus*) protein for 11 consecutive weeks on muscle mass and muscle strength in older adults.

**Methods:**

In this randomized controlled trial, 70 physically active older adults (>60 years) were randomly allocated to three groups: (I) lesser mealworm protein, (II) whey protein or (III) iso-caloric placebo. Participants received 11 weeks of supplements two times a day (30 gram/day). Muscle mass, fat mass, leg muscle strength and handgrip strength were measured at baseline and after 11 weeks of supplementation.

**Results:**

Of the 70 participants, 59 completed the supplementation period (mealworm n = 16; whey n = 23; iso-caloric placebo n = 20). Overall, skeletal muscle mass increased from 29.0 ± 6.2 kg to 29.3 ± 6.1 kg, with a significantly more profound increase in the lesser mealworm group (+0.67 [0.20–1.14] kg) compared to the whey (+0.03 [-0.20 – 0.28] kg) and placebo group (+0.30 [0.03 – 0.63] kg, P_group*time_ = 0.030). Fat mass and maximum handgrip strength decreased over time, whereas one-repetition maximum (1RM) leg muscle strength did not change pre- versus post-intervention. No group differences, nor interaction effects, were observed for fat mass, leg muscle strength and handgrip strength

**Conclusion:**

11-weeks of lesser mealworm protein supplementation induced an increase in skeletal muscle mass compared to whey protein supplementation and iso-caloric placebo in physically active older adults. No differences among groups were observed for changes in muscle strength.

## 1 Introduction

Sarcopenia, known as age-related progressive loss of muscle mass and strength, is one of the most important contributors to disability in older adults [1]. The maintenance of skeletal muscle mass is directly affected by the amount of daily protein intake [2]. Older adults have a higher anabolic threshold for protein, also known as anabolic resistance [3]. Therefore, they need a higher protein intake for optimal muscle protein synthesis (MPS) compared to younger adults [4]. Unfortunately, more than 50% of active older adults do not reach the threshold of 1.2 gram/kg protein per day [5]. Several randomized controlled trails have shown positive effects of whey and milk-based protein supplements, with or without concurrent resistance training, on muscle characteristics in older adults [[6], [7], [8]].

The world’s increasing population challenges the availability of sufficient and high-quality dietary protein resources in the future. Insects might be a promising protein alternative to milk-based proteins since its well-balanced amino acid composition meets the essential amino acid (EAA) requirements and ingestion of lesser mealworm showed an increase of EAA in the blood circulation [9,10]. Previous literature showed that one dose of mealworm protein supplementation is able to increase MPS, both at rest and during recovery from exercise [11]. However, the value of long-term insect-based protein supplementation, such as lesser mealworm (*Alphitobius diaperinus)*, on body composition and muscle characteristics has not been examined yet.

This randomized double-blind placebo-controlled trial aimed to assess the effect of 11 weeks of lesser mealworm versus whey protein supplementation and iso-caloric placebo on skeletal muscle mass and muscle strength. We hypothesized that lesser mealworm and whey protein supplementation would be able to increase muscle mass and -strength, compared to the iso-caloric placebo.

## 2 Methods

### 2.1 Participants

Recruitment was initiated by sending emails to participants of the Nijmegen Exercise Study database who had opt-in for follow-up studies [12]. Individuals aged ≥60 years, who registered for the Nijmegen Marches 2022 (the largest walking events in the world, https://www.4daagse.nl/en) were eligible for inclusion. Exclusion criteria were (I) allergy for milk proteins, lactose, shell or shellfish, (II) a BMI > 30 kg/m^2^, (III) a diagnosis of Chronic Obstructive Pulmonary Disease (COPD), renal insufficiency, or intestinal diseases that may influence protein uptake, or (IV) cancer treatment or use of statins. Moreover, consumption of other protein supplements or performance of resistance exercise training was not allowed during the study period. All participants provided written informed consent prior to any experimental procedure. The study conformed to the principles of the Declaration of Helsinki, was approved by the local Medical Ethical committee (Study-ID: NL79716.091.21) and registered at the at the Dutch trial registry (NL9862).

### 2.2 Study design

**Fig. 1:**
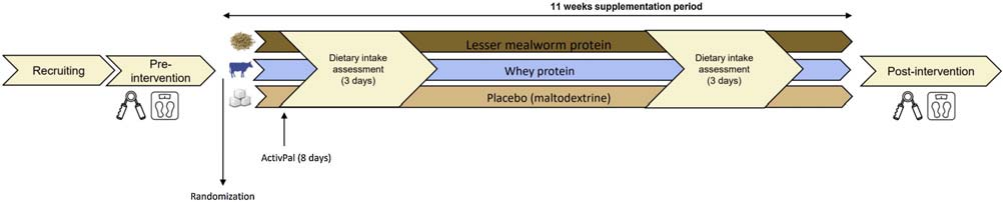
Study Design. Overview of study timeline and measurements. Hand-grip muscle strength, leg-press muscle strength and body composition were measured pre- and post-intervention.

This is a secondary analysis of a randomized double-blind placebo-controlled trial examining the effect of mealworm protein supplementation on muscle damage markers after prolonged walking exercise [13]. The sample size calculation was based on the primary outcome, and we recruited a total of 70 participants. Participants were invited for two study visits. Measurements were performed pre-intervention (upon randomization) and after 11 weeks of protein supplementation (post-intervention) (Fig. 1).

### 2.3 Randomization and masking

Directly after enrolment, the researcher randomly allocated the participants into a lesser mealworm protein, whey protein or iso-caloric placebo supplement group (1:1:1) using a computerized algorithm (Castor, Electronic Data Capture 2021, Ciwit B.V., Amsterdam, The Netherlands). Randomization was stratified for age, gender and registered walking distance for the Nijmegen Marches to ensure balance of the supplementation arms. The supplementation sachets were masked with numbers (A01, A02 and A03). Only the manufacturer had the key, the research team was blinded and unblinding took place after all the result of the study were available and were locked in the database (Castor, Electronic Data Capture 2021, Ciwit B.V., Amsterdam, The Netherlands).

### 2.4 Protein intervention

Participants were instructed to consume the assigned supplement every day for a period of 11 subsequent weeks (May–July 2022). The supplements were provided as a dried powder in blinded and labelled sachets with unique charge numbers. The supplement powder had a chocolate-coconut taste and had to be dissolved in a liquid (e.g., water, juice). Participants were instructed to consume one sachet in the morning and another sachet in the afternoon or after physical exercise, resulting in a total of 31 grams, 32 grams and 3 grams additional protein intake per day for respectively the lesser mealworm (*Alphitobius diaperinus)*, whey and placebo group (Table 1). The small amount of protein in the placebo supplement was due to the coconut flakes in the powder. All supplements were supplied by YNSECT Nutrition & Health (Ermelo, The Netherlands). Protein supplements were produced according to the HACCP/ISO22000 regulations at certified companies and were made from approved and commercially available ingredients.

**Table 1:** Nutritional composition of lesser mealworm protein, whey protein and placebo supplements.

Nutrient	Lesser mealworm Protein	Whey Protein	Maltodextrin (Placebo)
Energy (kcal/100 g)	467	463	491
Fat (g/100 g)	26.3	23.5	27.0
Carbohydrate (g/100 g)	6	14.2	54.0
Of which sugar (g/100 g)	2.2	4.3	4.9
Protein (g/100 g)	48.0	47.5	4.2
Fiber (g/100 g)	8.0	9.3	9.9
Amino Acid content (mg/100 g)			
Alanine	3100	2800	10
Arginine	2500	1300	15
Aspartanic Acid	3800	5800	23
Cystine	400	1300	4
Glutamic Acid	5700	9200	38
Glycine	2100	900	10
Histidine	1400	900	3
Isoleucine**	1900	3600	9
Leucine**	3000	3600	14
Lysine	3000	5100	8
Methionine	600	1100	3
Phenylalanine	1900	1600	11
Proline	3000	3300	11
Serine	1900	3000	12
Threonine	1800	3900	10
Tryptophane	500	800	3
Tyrosine	3000	200	3
Valine**	2500	3400	14

** Branches Chain Amino Acids (BCAA’s).

### 2.5 Measurements

#### 2.5.1 Anthropometrics and muscle mass

Height, weight, skeletal muscle mass and waist- and hip circumference of the participants were measured pre- and post-intervention. Waist-hip ratio was subsequently calculated. Total skeletal muscle mass (SMM) and Fat mass (FM) were assessed with bioelectrical impedance analyses (InBody 770 Body Composition Analyzed, Seoul, South Korea) [14]. Participants were instructed not to eat or drink for 2 h prior to the measurement and were asked to empty their bladder shortly before their body composition measurement.

#### 2.5.2 Muscle strength

Handgrip strength of the right hand was measured with a hydraulic, analogue handheld dynamometer (JAMAR®, Chicago, IL, USA). For every participant, the dynamometer was adjusted to their hand size at the first assessment, and the same dynamometer apparatus and adjustments were used for the follow-up measurements. The participants were seated in a chair with the elbow flexed in a 90-degree angle position. Arm support by chair was not allowed. Participants were asked to shortly squeeze the handgrip instrument as hard as they could for three times, with one minute rest in between each measurement [15]. Maximum strength in kilograms was used for analysis.

Leg strength was measured with an EN-Dynamic seated leg press (Enraf-Nonius, Rotterdam, The Netherlands). After adjusting the seat and a warm-up (10 repetitions (reps) with 50% bodyweight), participants had to perform 8–12 reps starting with 150% of the individual bodyweight. If the participant was able to perform more than 12 reps, the weight was raised by 15−35 kg according to standard operating procedures. If the participant was not able to perform more than 7 reps, the weight was lowered by 5 kg. Rest between the sets was 1 min and the maximum of attempts was 4. Based on the weight that was reached during the last attempt, the one-repetition maximum (1RM) was calculated using the Brzycki formula and corrected for bodyweight [16].

#### 2.5.3 Habitual physical activity

Habitual physical activity behavior was measured upon randomization with the ActivPAL accelerometer (PAL technologies Ltd, Glasgow, UK) [17]) for 8 consecutive days. The monitor is a small device (25 × 45 × 5 mm), attached to the upper thigh. A self-reported sleep diary in combination with a validated algorithm was used to identify sitting, standing and stepping during wear-time [18]. A measurement day was considered invalid when I) a single activity took up more than 95% of total awake time, II) daily step count was below 1,000 or III) the number of awake hours was less than 10. A valid assessment consisted of ≥5 valid days, including at least 1 weekend day [19]. Outcomes were reported as sitting time (hours/day), step count (number/day), time spent in light intensity physical activity (LIPA, min/day) and time spent in moderate-to-vigorous physical activity (MVPA, min/day) [19]. Cumulative walking distance was assessed by a weekly questionnaire that was sent out during the supplementation period. Study participants completed questions on the number of walking session and the distance per session. Accordingly, cumulative walking distance was calculated.

#### 2.5.4 Compliance and complaints

To assess the compliance, participants were asked to report their supplement intake every day by filling in a diary. Compliance was calculated by the consumed portions divided by the total portion they had to take during the 11-week supplementation period. Complaints were assessed using a questionnaire at week 4, 8 and 12. Questions consisted of changes in stool, loss of appetite, nausea, and stomach aches.

#### 2.5.5 Dietary intake

Food consumption patterns were assessed before and in the last week of supplementation period using an online Dutch tool (i.e., ‘*Mijn Eetmeter)* [20]. Participants entered their full eating and drinking pattern twice for 3 consecutive days, with at least one weekend day within this timeframe. The nutritional composition of the supplement was manually added to each dietary intake file by the researcher after unblinding. The average total energy intake, as well as the protein intake were subsequently calculated.

## 3 Statistical analysis

Statistical analyses were performed using SPSS software (IBM SPSS Statistics for Windows, Version 25.0 IBM Corp., Armonk, NY, USA) and graphs were made using Graphpad Prism 9. A per protocol analysis was used to assess main effects. All continuous variables and the residuals of the variables used in the linear-effects model were visually inspected and tested for normality with the Kolmogorov-Smirnov test. Data were displayed as mean ± SD or median (interquartile range [IQR]) for parametric and non-parametric continuous variables, respectively. Group differences at baseline were analyzed using a One-Way ANOVA or Kruskal Wallis for parametric or non-parametric continuous variables, respectively. Repeated observations, such as changes in body composition and muscle strength, were analyzed using repeated measures ANOVA which took baseline and post-intervention data of all groups into account and provided P-values for time, group and interaction effects.

When significant main effects or interactions were detected, Bonferroni post-hoc comparisons were made in case of parametric variables and Mann–Whitney U tests in case of non-parametric variables. The incidence of drop outs in the whey and lesser mealworm group was compared between the placebo group and the odds ratios were calculated using regression analyses. The incidence of dropout of the lesser mealworm group and whey group was compared with the placebo group. The level of significance was set at p < 0.05 (two-sided).

## 4 Results

### 4.1 Participants

**Fig. 2:**
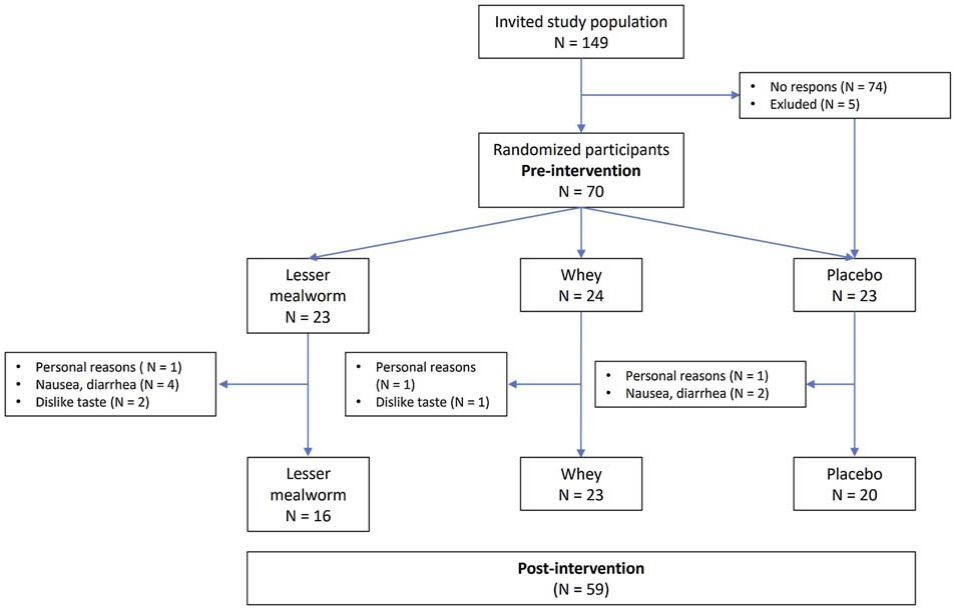
Flow chart with drop-outs. The reasons for discontinuation of supplementation were due to physical symptoms, dislike of the supplement taste and personal reasons not related to the supplement. The analytical cohort contains 59 participants.

A total of 70 participants were recruited (Supplemental Table S1) of which 59 participants completed the 11-week supplementation period (mealworm n = 16; whey n = 23; placebo n = 20). Dropout rates did not differ between the lesser mealworm group (OR: 2.9 [95% CI 0.65–13.12]; p = 0.16), whey protein group (OR: 0.29 [95% CI 0.028–3.01]; p = 0.16) and placebo group (reference group). The reasons for discontinuation of supplementation was due to I) physical symptoms (lesser mealworm n = 4; placebo n = 2) such as diarrhea, loss of appetite, nausea and floated stomach feeling, II) dislike of the supplement taste (lesser mealworm n = 2; whey n = 1) and III) personal reasons not related to the supplement (lesser mealworm n = 1; whey n = 1) (Fig. 2). Participants of the analytical cohort (n = 59, 51% male) were 69 ± 5 years old, with a BMI of 24.6 ± 2.7 kg/m^2^, no major differences in baseline demographics or anthropometrics were found across groups (Table 2).

**Table 2:** Pre-intervention characteristics of the analytical cohort and specified for the mealworm--, whey- and placebo supplement group.

	Total group n = 59	Lesser mealworm n = 16	Whey n = 23	Placebo n = 20	p-value
Demographics					
Age (years)	69 ± 5	68 ± 4	70 ± 5	68 ± 5	0.25
Male, n (%)	30 (51)	9 (56)	12 (52)	9 (45)	0.79
Anthropometrics					
Body weight (kg)	72.9 ± 12.9	76.8 ± 14.2	72.9 ± 12.3	69.7 ± 12.3	0.26
Height (m)	1.72 ± 0.98	1.72 ± 0.10	1.72 ± 0.98	1.70 ± 0.97	0.81
BMI (kg/m^2^)	24.6 ± 2.7	25.9 ± 3.0	24.4 ± 2.3	23.8 ± 2.7	0.05
Waiste-hip ratio	0.93 ± 0.09	0.97 ± 0.09	0.95 ± 0.09	0.89 ± 0.08	**0.035**
Skeletal muscle mass (kg)	29.4 ± 6.2	29.1 ± 6.5	29.7 ± 6.3	28.3 ± 6.0	0.72
Fat mass (kg)	19.1 ± 5.4	20.7 ± 6.3	19.0 ± 5.1	18.0 ± 4.4	0.33
Physical activity*					
Sedentary time (h/day)	9.0 ± 1.8	9.4 ± 2.2	9.1 ± 1.3	8.4 ± 2.0	0.35
Step count (n/day)	7336 [5993−9369]	6599 [5678−9537]	7444 [6005−9004]	7502 [5744−10263]	0.82
MVPA (min/day)	115 [94−145]	108 [86−145]	115 [96−147]	121 [85−159]	0.85
LIPA (min/day)	259 [193−335]	267 [182−350]	257 [192−304]	250 [196−367]	0.89
Cumulative walking distance during supplementation period (km)	460 [323–671]	462 [346−742]	594 [395−729]	450 [335−761]	0.47

Data are presented as number (with percentage between brackets) of participants, mean ± SD for parametric data or median [interquartile range] for non-parametric data. Body Mass Index (BMI), h = hours. N = number; min = minutes; MVPA = moderate to vigorous physical activity; LIPA = light intensity physical activity; km = kilometer.

* ActivPal measurement failed for 8 participants due to an error in the system.

### 4.2 Physical activity

Eight ActivPAL measurements were missing due to devices’ error, leaving data of 55 participants available. Participants spent 115 [94−145] min/day MVPA and 259 [193−335] min/day LIPA, step count was 7,336 [5,993−9,369] and sedentary time was 9.0 ± 1.8 h per day. No differences among groups were observed for MVPA, LIPA, step count and sedentary time (p = 0.85, p = 0.89 and p = 0.82, p = 0.35, respectively (Table 2). The cumulative walking distance during the supplementation period was 460 [323–671] kilometers, which was comparable across groups (p = 0.53).

### 4.3 Compliance and complaints

The supplement intake compliance was 98% [96–100], 99% [98–100] and 100% [98–100] for the lesser mealworm, whey and placebo group, and did not differ among the groups (P_group_ = 0.11). 35 out of 59 (59%) participants reported gastro-intestinal symptoms which could be related to the supplement (nausea, change in stool, decreased appetite and stomach aches, Table 3). Change in stools was the most common symptom (n = 26, 44%) followed by decreased appetite (n = 16, 27%). The whey protein group showed a significant higher rate for change in stools than the lesser mealworm and placebo group (p = 0.035).

**Table 3:** Complains during the supplementation period.

	Total group N = 59	Lesser mealworm N = 16	Whey N = 23	Placebo N = 20	p
Symptoms in general (N)	35 (59)	8 (50)	17 (73)	10 (50)	0.19
Nausea (N)	5 (9)	2 (13)	2 (9)	1 (5)	0.72
Stomach aches (N)	8 (14)	2 (13)	3 (13)	3 (15)	0.97
Decreased appetite (N)	16 (27)	5 (31)	5 (22)	6 (30)	0.76
Change of stools (N)	26 (44)	5 (31)	15 (65)	6 (30)	**0.035**

Number of participants with percentage (%) between brackets.

### 4.4 Protein intake

**Fig. 3:**
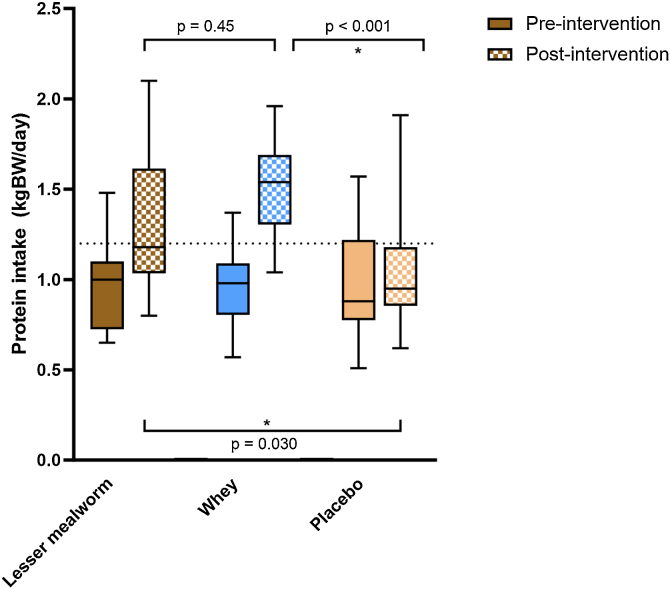
Protein intake. Box-and-whisker plots for protein intake. The box-and-whisker plots represent the median, interquartile range, 5-95% percentile (upper and lower whiskers). Mean protein intake significantly increased in the lesser mealworm (p < 0.05) and whey protein (p < 0.05) supplementation group from pre- to post-intervention whereas no changes occurred in the iso-caloric placebo group (p = 0.45).

Eight of the 59 participants did not fill in the dietary intake diaries. Protein intake increased from 0.99 ± 0.28 to 1.36 ± 0.42 g/kg/d in the lesser mealworm group and 0.96 ± 0.13 to 1.52 ± 0.27 g/kg/d in the whey group. Protein intake in the placebo group did not change (0.96 ± 0.3 g/kg/d at pre-intervention versus 1.05 ± 0.30 g/kg/d post intervention; (Fig. 3)). The prevalence of participants with a protein intake >1.2 g/kg/day increased from 23% to 69% in the lesser mealworm group and from 20% to 90% in the whey protein group, but remained 29% in the placebo group.

### 4.5 Body composition

**Fig. 4:**
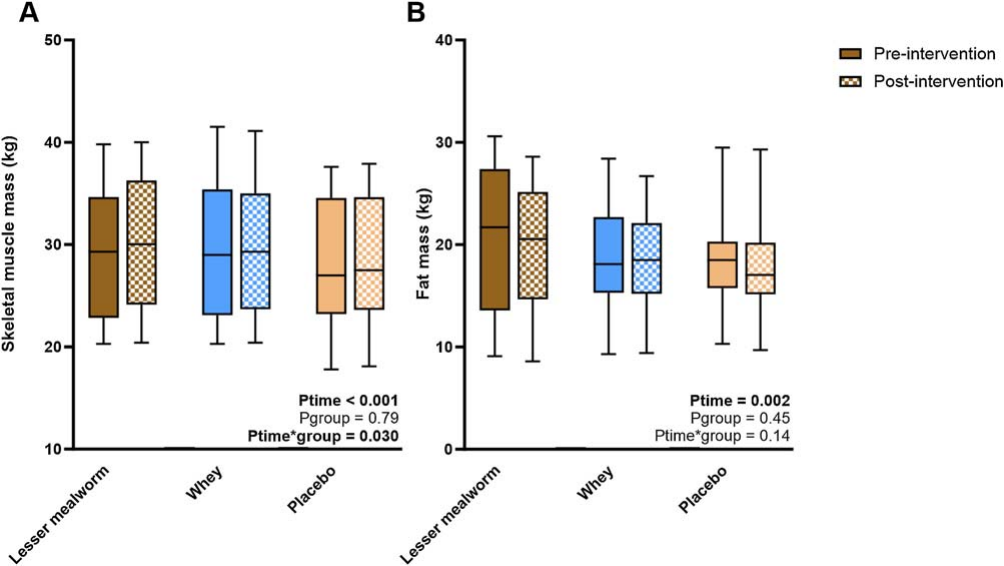
Skeletal muscle mass & fat mass. Box-and-whisker plots for skeletal muscle mass and Fat Mass. The box-and-whisker plots represent the median, interquartile range, 5-95% percentile (upper and lower whiskers). The increase in SMM was more profound in the lesser mealworm protein group compared to the whey protein group and the placebo group. Reductions in FM did not change across groups.

Body weight did not change from pre- to post-intervention in any of the groups (P_time_ = 0.48; P_group*time_ = 0.32). Overall, SMM increased from 29.0 ± 6.2 kg to 29.3 ± 6.1 kg (P_time_ <0.001), with different responses across groups (P_group*time_ = 0.030). The increase in skeletal muscle mass was more profound in the lesser mealworm group (+0.67 [0.20–1.14] kg) compared to the whey (+0.03 [-0.20 – 0.28] kg) and placebo group (+0.30 [0.03 – 0.63] kg). FM decreased from 19.1 ± 5.4 kg at pre-intervention to 18.5 ± 5.1 kg at post-intervention (P_time_ = 0.002), with comparable responses across groups (P_group*time_ = 0.14, Fig. 4).

### 4.6 Muscle strength

**Fig. 5:**
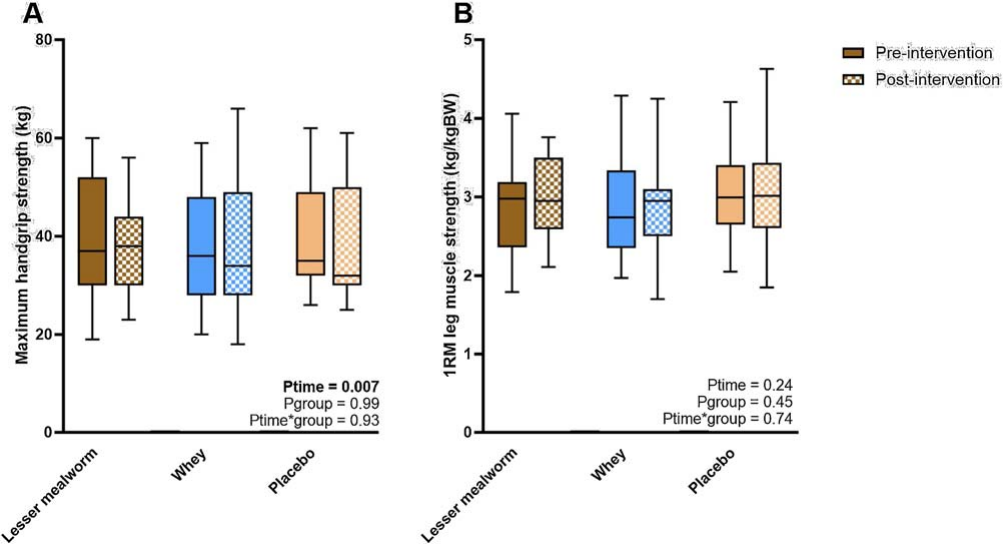
Handgrip strength and leg press strength. Box-and-whisker plots for maximum handgrip strength (A) and 1RM leg muscle strength corrected for bodyweight (B). The box-and-whisker plots represent the median, interquartile range, 5–95% percentile (upper and lower whiskers). Both values in maximum handgrip strength and 1RM leg muscle strength corrected for bodyweight did not differ among groups.

Maximum handgrip strength decreased from 39.3 ± 11.0 kg at pre-intervention to 37.7 ± 11.4 kg at post-intervention (P_time_ = 0.007, Fig. 5), whereas 1RM leg muscle strength corrected for bodyweight did not change (P_time_ = 0.24). No group differences were observed for changes in handgrip strength (P_group*time_ = 0.99) and leg strength (P_group*time_ = 0.74) (Fig. 4A & B).

### 4.7 Discussion

To the best of our knowledge, this is the first human study to assess the effect of prolonged lesser mealworm protein supplementation on changes in muscle characteristics in physically active older adults. We found an increased SMM following supplementation and this increase was more profound in the lesser mealworm group compared to whey protein and the placebo group. These findings indicate that 11 weeks of lesser mealworm supplementation exhibit positive effects on muscle mass in physically active older adults. In all three groups, FM and maximum handgrip strength decreased from the pre- to post-intervention, while normalized 1RM leg muscle did not. None of these responses differed across groups.

Eleven weeks of lesser mealworm protein supplementation induced an increase in skeletal muscle mass in older adults, confirming our hypothesis. Lesser mealworm-derived proteins consists of essential and non-essential amino-acids, and previous studies have shown that ingestion of lesser mealworm protein isolate enhances blood amino acid concentrations comparable to soy protein [10,11]. Adequate levels of amino acids are required to induce muscle protein synthesis [[21], [22], [23]]. These findings reinforce animal experiments, showing that lesser mealworm derived protein supplementation can attenuate skeletal muscle atrophy in hindlimb casting immobilized rats [24]. In contrast, a study in young athletes did not find changes in body composition after eight weeks of lesser mealworm supplementation [25]. Possible explanations for these contradictory findings may relate to the fact that these athletes were already on a high protein diet before study enrolment, and/or that they were well trained and subsequently less room to improve body composition.

The superior increases in skeletal muscle mass for the lesser mealworm supplementation group compared to the whey protein group is remarkable since previous literature already showed positive effects of whey protein supplementation on body composition [26], given its well-balanced amino acid composition [27] (Table 1). The different responses between the lesser mealworm protein group and whey protein group may be attributable to small, but non-significant, differences in participant characteristics as the mealworm group was younger, had lower habitual physical activity levels at baseline, and a higher cumulative walking distance during the supplementation period compared to the whey protein group. The combination of these factors could make them more disposed to gain muscle mass [28].

Among our secondary outcomes, handgrip strength decreased while normalized 1RM leg muscle strength did not change over time. These findings are somewhat unexpected and contradictory to a recent meta-analysis reporting improvements in handgrip strength following whey protein supplementation in combination with resistance exercise training but not following protein supplementation alone [29]. Although our study population was physically active, walking is aerobic exercise and participants did not follow a specific resistance exercise training program. Additional studies on the effects of lesser mealworm and other protein supplementations on muscle function are, therefore, warranted.

The strength of this study was that this is the first study of prolonged mealworm supplementation on SMM and muscle strength. However, some limitations should be considered. First, the prevalence of drop out was relatively high (16%). Nevertheless, the power of the study was sufficient to show differences in muscle outcomes, and the drop-out rate did not differ across groups. Second, we recruited a physically active study population, highlighted by 115 min/day of MVPA, as the combination of a physically active lifestyle and sufficient protein intake is required for maintaining or gaining muscle mass [26,30,31]. However, this approach may attenuate the generalizability of our findings to a less active or sedentary population. At last, protein supplementation was not adjusted to body weight, which means that all participants consumed the same amount and this might have resulted in over- and underdose of the supplement depending on the individuals body weight. However, body weight was not different between groups, so it is unlikely that this has affected our outcomes. Future studies may consider to adjust the dosage of the protein according to the body weight of the participant to optimize the effect and decrease potential (health) complains.

In conclusion, 11 weeks of lesser mealworm protein supplementation induced a greater increase in skeletal muscle mass compared to whey protein supplementation and placebo in physically active older adults. These findings highlight the potential of insect proteins to target sarcopenia in older adults with a physically active lifestyle.

## Conflicts of interest

The results of the study are presented clearly, honestly, and without fabrication, falsification, or inappropriate data manipulation. The authors declare no conflict of interest.
