# Supplementary material for: Daily supplementation of lesser mealworm protein for 11-weeks increases skeletal muscle mass in physically active older adults
Source: J Nutr Health Aging. 2024 Sep 21;28(11):100364. doi: 10.1016/j.jnha.2024.100364 (PMC12879208; doi:10.1016/j.jnha.2024.100364)
Supplement: Supplementary file 1 [file mmc1.docx]

**Supplemental table 1| Pre-intervention characteristics of the total recruited group and specified for the whey-, Mealworm- and placebo supplement group.**

|  | Total group  N = 70 | Mealworm  N = 23 | Whey  N = 24 | Placebo  N = 23 | p-value |
| --- | --- | --- | --- | --- | --- |
| Demographics |  |  |  |  |  |
| Age (years) | 69 ± 5 | 68 ± 4 | 70 ± 5 | 68 ± 5 | 0.16 |
| Male (%) | 33 (47) | 10 (44) | 12 (52) | 12 (52) |  |
| Anthropometrics |  |  |  |  |  |
| Body weight (kg) | 72.4 ± 14.0 | 76.8 ± 14.2 | 72.9 ± 12.3 | 71.8 ± 15.1 | 0.97 |
| Height (m) | 1.71 ± 0.96 | 1.72 ± 0.10 | 1.72 ± 0.98 | 1.72 ± 0.10 | 0.70 |
| BMI (kg/m^2^) | 24.6 ± 3.1 | 25.9 ± 3.0 | 24.4 ± 2.3 | 24.1 ± 3.0 | 0.51 |
| Waist-hip ratio | 0.92 ± 0.10 | 0.93 ± 0.10 | 0.93 ± 0.10 | 0.89 ± 0.09 | 0.32 |
| Skeletal muscle mass (%) | 29.0 ± 6.3 | 28.4 ± 6.6 | 29.4 ± 6.4 | 29.0 ± 6.3 | 0.90 |
| Dietary intake* |  |  |  |  |  |
| Energy intake (kcal) | 1765 ± 399 | 1800 ± 339 | 1751 ± 411 | 1747 ± 458 | 0.90 |
| Protein intake (g/kg/d) | 0.96 ± 0.25 | 0.96 ± 0.26 | 0.96 ± 0.19 | 0.97 ± 0.31 | 0.99 |
| Number of participants with a protein intake >1.2 g/kg/d (%) | 9 (16) | 2 (13) | 2 (9) | 5 (26) | 0.29 |
| Physical activity** |  |  |  |  |  |
| Sedentary time (h) | 9.0 ± 1.8 | 9.3 ± 2.2 | 9.1 ± 1.3 | 8.4 ± 2.0 | 0.35 |
| Step count (n) | 7408  [5993-9251] | 6749  [5664-9274] | 7444  [6005-9003] | 7596  [5946-9926] | 0.82 |
| MVPA (min) | 116 [94-145] | 110 [87-143] | 115 [96-147] | 122 [88-158] | 0.85 |
| LIPA (min) | 255 [194-326] | 260 [197-327] | 257 [192-304] | 248 [197-360] | 0.89 |
| Cumulative walking distance during supplementation period | 460  [323 – 671] | 462  [346-742] | 594  [395-729] | 450  [335-761] | 0.47 |

Data are presented as number (with percentage between brackets) of participants, mean $\pm$ SD. Body Mass Index (BMI), Skeletal Muscle Mass (SMM (%)), percentage skeletal muscle mass, calculated by (skeletal muscle mass / the participant’s bodyweight) * 100. h = hours. n = number; min = minutes; MVPA = moderate to vigorous physical activity; LIPA = light intensity physical activity.
